# Beyond minutes played: Rotation architecture as a persistent club-level signature in elite basketball

**DOI:** 10.1371/journal.pone.0360790

**Published:** 2026-10-27

**Authors:** Jaime Sampaio, Miguel Ángel Gómez, Francesco Cuzzolin, Julio Calleja-González

**Affiliations:** 1 Research Center in Sports Sciences, Health Sciences and Human Development (CIDESD), University of Trás-os-Montes and Alto Douro (UTAD), Vila Real, Portugal; 2 Performance Advisory Board, EuroLeague Players Association (ELPA), Barcelona, Spain; 3 Facultad de Ciencias de la Actividad Física y del Deporte, Universidad Politécnica de Madrid, Madrid, Spain; 4 UCAM, Catholic University of Murcia, Murcia, Spain; 5 Department of Physical Education and Sports, Faculty of Education and Sport, University of the Basque Country (UPV/EHU), Vitoria-Gasteiz, Spain; Universidade Federal de Goias, BRAZIL

## Abstract

Basketball substitutions are usually interpreted as tactical decisions, but repeated substitution sequences may also reveal stable team-level tendencies in how competitive exposure is organised. We reconstructed 65,657 on-court stints from two complete EuroLeague regular seasons and defined a proposed five-indicator team rotation signature (S5), evaluated over two seasons: structural exposure per minute, fragmentation, rotation irregularity, compressed recovery, and fourth-quarter SEI share. Game-level signatures were identifiable above chance, with leave-one-out nearest-centroid accuracies of 26.7% (95% CI [23.2, 30.0]) and 31.1% (95% CI [27.8, 34.5]) against chance levels of 5.6% and 5.0%, and noisy game-level profiles aggregated into reliable seasonal fingerprints. Across the 17 clubs present in both seasons, matched clubs remained closer to their own previous signature than to randomly paired club-season profiles (mean distance 1.68 vs. 2.62; p < .001). Game state affected rotation depth far more than the signature: a 20-point margin explained 14.33% of within-club variance in effective rotation but 0.80% in signature deviation. Structural consolidation was not detectably associated with winning. However, clubs concentrating structural exposure in the fourth quarter won fewer games (r = −0.38, 95% CI [−0.66, −0.05]); this exploratory association replicated in both seasons but attenuated after controlling for previous-season success and should not be read causally. Rotation architecture therefore behaves as a comparatively persistent club-level property rather than a by-product of in-game substitution decisions.

## Introduction

Performance analysis in team sports has long relied on indicators that summarise competitive behaviour, identify stable performance profiles and support coaching decisions [1,2]. Yet a persistent challenge is that game-level indicators are noisy. A single match may reflect opponent strength, score line, tactical adjustments, fatigue, officiating style and chance. For this reason, performance profiles are only useful if they distinguish transient game-state variation from more persistent tendencies in how players and teams organise behaviour [3,4].

Basketball is a particularly suitable setting because rotation is central to coaching decisions and game dynamics, and substitution is both frequent and consequential: coaches change line-ups repeatedly, manage foul trouble, alter matchups, protect players, and respond to the scoreboard. Previous research has shown that substitutions influence local game events [5], that rotation can be represented through substitution networks [6], and that players or teams can be profiled through game-related statistics and contemporary modelling [7–9]. Contextual factors such as score line, game location, opponent quality and schedule congestion also shape game behaviour and performance indicators [10–13]. However, most available descriptions remain centred on how many players are used, how many minutes they play, or how substitution events are connected. Less is known about the temporal architecture through which playing exposure is assembled. Two teams may allocate the same minutes and use a similar number of players yet expose athletes through different temporal structures: long uninterrupted stints, many short fragmented ones, irregular sequences, compressed recovery, or disproportionate late-game load. Such structural differences may plausibly bear on fatigue, attention, role continuity, tactical rhythm and the distribution of load across a game [14]. From a complex-systems perspective, they may also reveal organisational tendencies that are noisy locally but stable cumulatively: repeated local decisions need not be identical for a team-level pattern to be recognisable [15–19].

We use the term rotation architecture for this temporal organisation of competitive exposure, intentionally distinct from rotation depth. Depth describes how many players are used; architecture describes how exposure is structured through stint intensity, fragmentation, irregularity, recovery compression and temporal placement. The pivotal question is therefore not whether coaches rotate, but whether the way they assemble exposure behaves as a stable team-level signature.

The primary aim of this study was to develop and evaluate a parsimonious, stint-based framework for describing how elite basketball clubs structure competitive exposure, operationalised as a five-indicator rotation signature reconstructed from two EuroLeague seasons. Within that framework, we then addressed three secondary questions: whether the signature is identifiable, reliable and persistent across scales; whether it is less responsive to game state than rotation depth; and whether its structural and temporal aspects are associated with competitive success. We expected rotation depth to respond strongly to the scoreboard, but rotation architecture to show stronger aggregation, persistence and club-level structure.

## Materials and methods

### Data source and stint reconstruction

Play-by-play substitution records were obtained for two complete EuroLeague regular seasons, 2024−25 and 2025−26, comprising 18 and 20 competing clubs, respectively. For every player in every game, court entries and exits were paired to reconstruct stints, defined as uninterrupted intervals of on-court participation. The reconstruction yielded 65,657 stints.

Reconstruction fidelity was verified against the regulation structure of the game. Total on-court team time per game should equal five players multiplied by 40 minutes, that is, 200 minutes. The observed median was exactly 200 minutes per team-game, and only 0.14% of team-games fell outside a 190–230 minute window; every such case was attributable to overtime. Terminal stints, which have no observable subsequent rest interval within the same game, were excluded from recovery calculations, leaving 50,370 stints with a defined recovery ratio.

### Multiscale analytical design

Rotation was analysed at nested scales. The stint scale recorded individual exposure events. The player-game scale aggregated a player’s stints within a game. The player-season scale aggregated across games. The team-game scale aggregated across the players of a team within a game. The team-season scale aggregated across games of a season and constituted the unit at which the final team rotation fingerprint was defined.

All inferential claims were made at the team-game or team-season scale. Stints and player-games are nested within players, teams and games and were therefore used to construct higher-scale indicators rather than treated as independent observations.

### Rotation indicators

Stints were classified by duration into four categories: micro (< 1 min), short (≥ 1 and < 3 min), moderate (≥ 3 and < 7 min) and long (≥ 7 min). The seven-minute boundary for the long class reflects the intermittent, high-intensity demands of elite basketball, in which sustained on-court exposure is thought to accrue physiological and mechanical load non-linearly; comparative activity-demand studies motivate treating extended continuous exposure as a qualitatively distinct load category [14,20,21].The Structural Exposure Index (SEI) assigns each stint an exposure value that grows superlinearly with duration and more steeply for longer stints. For a stint of duration d seconds in class c, the base index is SEI₀ = w(c) × d^e(c), with w = 1.00, 1.10, 1.25 and 1.50, and e = 1.00, 1.05, 1.15 and 1.35 for micro, short, moderate and long stints, respectively.

Two contextual multipliers were applied. A sequence penalty of 1 + 0.05 × (r – 1) was applied to long stints, where r is the length of the current run of consecutive long stints. A multiplier of 1.05 was applied to stints occurring in the final quarter. SEI per minute was computed as the sum of contextual SEI divided by total minutes on court. These weights, exponents and multipliers were fixed a priori to encode three assumptions (that longer stint classes carry disproportionately greater structural exposure than duration alone implies, that prolonged uninterrupted runs accumulate additional exposure, and that fourth-quarter exposure carries greater competitive stakes) rather than being tuned to any outcome. Their influence was examined directly: a sensitivity analysis re-estimated club identifiability under alternative specifications (a fully linear index, ± 1-minute class thresholds, flattened exponents, and removal of both contextual multipliers), and club separation in the S5 space was preserved throughout (S1 Table).

Fragmentation (FI10) was defined as the number of stints per 10 minutes on court. Rotation irregularity (RII) was the mean of three standardised quantities: the standard deviation of stint durations, the mean absolute successive difference between consecutive stint durations, and Shannon entropy of the stint-class distribution. The rotation recovery ratio (RRR) was defined as the rest interval following a stint divided by the duration of the stint. Compressed recovery was the proportion of stints with RRR < 1.00; this unity threshold is an operational cut-off, and the indicator was near-identical under alternative cut-offs of 0.90 and 1.10 (cross-threshold correlations 0.985-0.995). Fourth-quarter SEI share was the proportion of contextual SEI accrued in the fourth quarter. Only regulation-period stints were retained for indicator construction. Accordingly, fourth-quarter SEI share refers to the proportion of contextual SEI accrued in the fourth regulation quarter relative to total contextual SEI across regulation periods.

Because contextual SEI already up-weights fourth-quarter stints by 5%, fourth-quarter SEI share is a strictly increasing transform of the corresponding share of base SEI, share = 1.05x/ (1 + 0.05x). The multiplier is applied identically to every club. It therefore cannot generate between-club differences: across the observed range, the rank order of clubs is preserved exactly, and the maximum divergence between the standardised values computed with and without the multiplier is 0.006 SD (Supplementary Note S1).

### Team rotation signature

The final team rotation signature, hereafter referred to as S5 because it comprises five retained indicators, was measured identically at team-game and team-season scale (Table 1).

**Table 1:** The five indicators constituting the final S5 team rotation signature.

Indicator	Operational definition	Construct captured
SEI per minute	Contextual Structural Exposure Index accrued per minute on court	Structural exposure: concentration of exposure in longer and sequenced stints
Fragmentation (FI10)	Number of stints per 10 minutes on court	Episode structure: how finely exposure is broken into stints
Irregularity (RII)	Mean of standardised stint-duration SD, mean absolute successive difference and stint-class entropy	Temporal irregularity: dispersion and unpredictability of exposure
Compressed recovery	Proportion of stints followed by rest shorter than the preceding stint (RRR < 1.00)	Recovery pressure: prevalence of incomplete rest
Q4 SEI share	Proportion of contextual SEI accrued in the fourth quarter	Temporal placement: when structural exposure is spent within the game

Candidates were admitted only if they satisfied two independent criteria: not redundant with a retained indicator by construction, and stable between seasons (Table 2). A redundant indicator carries no independent information; an independent but temporally unstable one is not a property of the club and would inject identity-unrelated variance. Two candidates illustrate both failures. The mean rest-to-work ratio was independent of the retained set (maximum |r| = 0.28) but did not persist (r = −0.17) and showed negligible between-team consistency (ICC(1) = 0.01). The median rest-to-work ratio was stable across seasons (r = +0.57) but nearly collinear with compressed recovery (|r| = 0.95). Neither could enter: the first is independent but not a club property, the second a club property but not independent.

**Table 2:** Candidate indicators excluded from the signature and the criterion each failed.

Candidate	Definition	Independence	Stability	Criterion failed
Long-stint exposure	Minutes in long stints/ total minutes	r = 0.999 with SEI per minute	–	Redundant by construction
Exposure-balance ratio	(Short + moderate)/ long minutes	Algebraic transform of long-stint exposure	–	Redundant by construction
Severe compression	Proportion of stints with RRR < 0.50	Nested subset of compressed recovery (r = 0.97)	–	Redundant by construction
Extended recovery (mean RRR)	100 × mean rest-to-work ratio	max |r| = 0.28 (independent)	r = −0.17	Fails temporal stability
Median RRR	Median rest-to-work ratio	max |r| = 0.95 (redundant)	r = +0.57	Fails independence
Core players’ minutes concentration	Share of minutes in the core rotation	max |r| = 0.76	r = −0.19	Fails both criteria
Role players differentiation distance	Core-periphery profile distance	max |r| = 0.53	r = −0.40	Fails temporal stability

Independence is the maximum absolute correlation with a retained indicator at team-season scale. Temporal stability is the Pearson correlation of the standardised club value across the 17 clubs present in both seasons. Redundancy by construction is a design exclusion and does not require the stability criterion.

### Statistical analysis

The signature was analysed directly as a standardised five-dimensional profile. The conventional anchors (game minutes and total stints) were retained as comparators in Fig 1.

**Fig 1:**
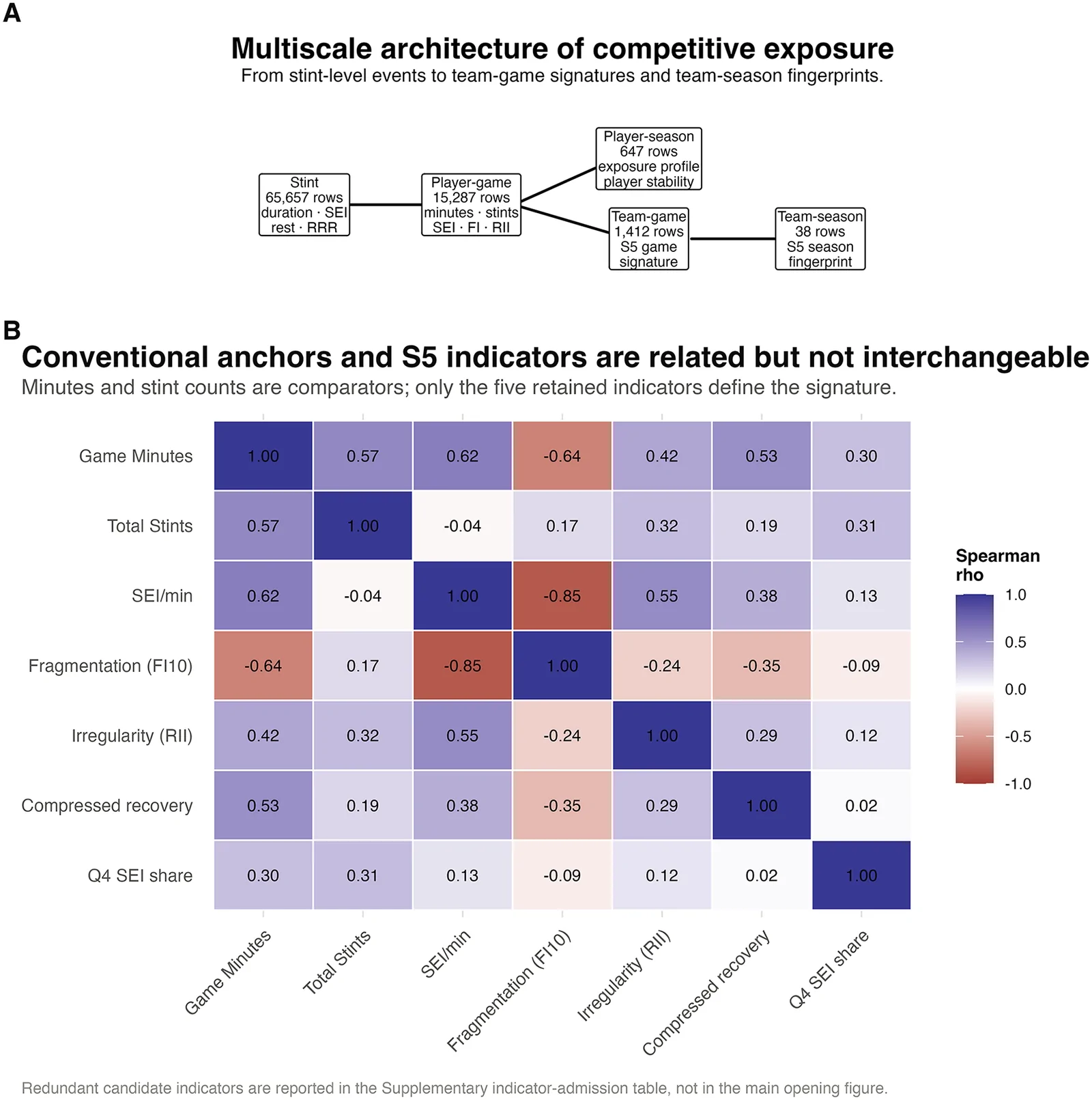
Multiscale architecture of competitive exposure and separation of conventional anchors from the final S5 signature. Panel A shows the analytical progression from stint-level events to team-game signatures and season-level fingerprints. Panel B shows Spearman correlations among game minutes, total stints, and the five retained S5 indicators. Game minutes and total stints are shown as conventional comparators only and are not part of the signature.

Within-season consistency was evaluated for each game-level indicator using one-way random-effects ICC(1). Split-half reliability was estimated by splitting each club’s games on the parity of the game index, computing indicator means within each half, and taking the Spearman rank correlation between halves across clubs. Contrasting ICC(1) with split-half reliability makes the Spearman-Brown expectation explicit: the aggregation gain is largest for the indicator with the lowest single-game consistency. Single-game identifiability was assessed by leave-one-out nearest-centroid assignment: each team-game was compared to the seasonal centroids of all clubs computed without that game. Standardisation was computed once within each season before leave-one-out assignment. Because each held-out game represented only 0.13-0.15% of the season-level standardisation sample, leakage through scaling parameters was considered negligible; team centroids were recomputed excluding the held-out game. Correct-assignment rates varied widely across clubs (0−100%; S2 Table), and this spread was itself structured rather than random: a minority of clubs carried highly distinctive rotation signatures recovered far above chance (in 2024−25, twelve of eighteen clubs exceeded twice the chance rate and seven exceeded five times chance), while others followed more conventional patterns indistinguishable from the league norm. This distinctiveness was persistent, with the most and least identifiable clubs tending to retain their status across seasons, so that average identifiability summarises a genuine gradient of rotational idiosyncrasy rather than a property held equally by all clubs. Two further analyses supported this interpretation. First, a crossed random-effects model (each standardised indicator modelled with club and season as crossed random intercepts) attributed a substantial share of indicator variance to the club: between-club repeatability ranged from 0.39 to 0.68 for four of the five indicators (structural exposure per minute 0.60, fragmentation 0.68, rotation irregularity 0.44, compressed recovery 0.39) and was lowest for fourth-quarter SEI share (0.11), consistent with the latter being the most tactically volatile dimension (S3 Table). Second, reduced representations were less identifiable than the full signature: rotation depth alone classified games at only 8.9% (barely above chance), a two-dimensional structural-plus-temporal representation reached 19.1%, and a structural pair (exposure per minute and fragmentation) reached 26.0%, against 28.9% for the full five-indicator signature (S4 Table). Rotation depth, the conventional descriptor, therefore carried almost none of the club-identifying information present in the architecture. Cumulative convergence was defined as the number of games required for a club’s cumulative fingerprint to fall within 0.5 standardised units of its final season fingerprint.

Between-season persistence was tested among the 17 clubs present in both seasons. Euclidean distance was computed between each club’s 2024−25 and 2025−26 standardised S5 profiles. A permutation test fixed first-season profiles and randomly reassigned the second-season club labels (B = 5,000). Sensitivity analyses repeated the test with a regularised Mahalanobis metric and after removing each indicator in turn.

An exploratory organisational-continuity analysis examined whether between-season S5 displacement differed according to head-coach continuity. For each of the 17 matched clubs, publicly documented head-coach sequences across 2024−25 and 2025−26 were reviewed. Clubs were classified according to whether they retained the same head coach throughout the observation window or experienced at least one coaching transition. The timing and sequence of all coaching changes are documented in S5 Table. Given the small, non-random sample and the post hoc nature of the analysis, inference was treated as exploratory. We report group medians and interquartile ranges, an exact Mann-Whitney test, an exact label-permutation test for the difference in medians, and a 5,000-sample bootstrap interval (seed 20260706).

Invariance to game state was tested by computing each team-game’s Euclidean deviation from its club’s leave-one-out seasonal fingerprint. Deviation was regressed on the absolute margin entering the fourth quarter and a binary indicator denoting whether the game was the second fixture of a EuroLeague double-game week. Club-season fixed effects absorbed stable between-club differences, and standard errors were clustered by club-season. Equivalent models were estimated for effective rotation and for the number of players used, as tactical contrast variables outside the signature. Effective rotation was defined as the effective number of players in the minute distribution, computed as the exponential of the Shannon entropy of each club’s share of minutes across players (so that a perfectly even distribution equals the roster size and a concentrated one approaches a smaller effective number). Schedule congestion and accumulated load were interpreted in relation to previous work on fixture load and fatigue risk [22].

Associations with success were examined at the team-season scale. Structural consolidation was defined a priori as the unit-weight average of four structural exposure indicators (SEI per minute, FI10, RII, and compressed recovery), excluding fourth-quarter load because it primarily reflects contextual end-game management rather than the underlying structure of player rotation. It was computed on standardised team-season values as [z(SEI per minute) – z(FI10) + z(RII) + z(compressed recovery)]/ 4, so that higher scores describe clubs sustaining longer, more irregular and less completely recovered stints. Unit weights involve no parameters fitted to the outcome and were fixed before any association with success was examined. Associations with win percentage and point differential were assessed using correlations, cluster bootstrap intervals resampling whole clubs (B = 5,000), and within-season permutation tests (B = 10,000) where appropriate.

All processing and analyses were conducted in R 4.6.0 using the tidyverse, ggplot2, and patchwork packages. A fixed random seed of 20260706 was used for all stochastic procedures, including permutation tests, cluster bootstrap resampling, and leave-one-out analyses, to ensure reproducibility of the reported intervals and p-values within the specified computational environment. Intraclass correlations were computed as one-way random-effects ICC(1) following Koo and Li [23], with negative estimates floored at zero. The regularised Mahalanobis metric used a shrinkage covariance estimator (λ = 0.10) of the class described by Ledoit and Wolf [24] and Schäfer and Strimmer [25].

### Sample size justification

The sample was the complete population of EuroLeague regular-season games across two seasons rather than an a priori sample. Effective inferential samples were 1,412 team-games, 1,366 with complete quarter scoring for game-state models, and 38 team-seasons; the 46 team-games without complete quarter scoring (23 games) all occurred in the 2024−25 season and were distributed across ten clubs, reflecting incomplete quarter-level data in the earlier season rather than any systematic exclusion; between-season persistence used the 17 clubs present in both seasons. Precision and stability were evaluated through split-half reliability, leave-one-game-out procedures, club-level bootstrap resampling and permutation tests.

## Results

### Game-level signatures are identifiable and stable within a season

Variance decomposition of the five game-level indicators revealed a consistent hierarchy (Fig 2). Load-structure indicators behaved most like team traits: fragmentation ICC = 0.70 and 0.78, and structural exposure per minute ICC = 0.66 and 0.71. Irregularity was intermediate (0.49 and 0.50), compressed recovery was season-dependent (0.26 and 0.52), and Q4 SEI share showed low single-game consistency (0.09 and 0.14).

**Fig 2:**
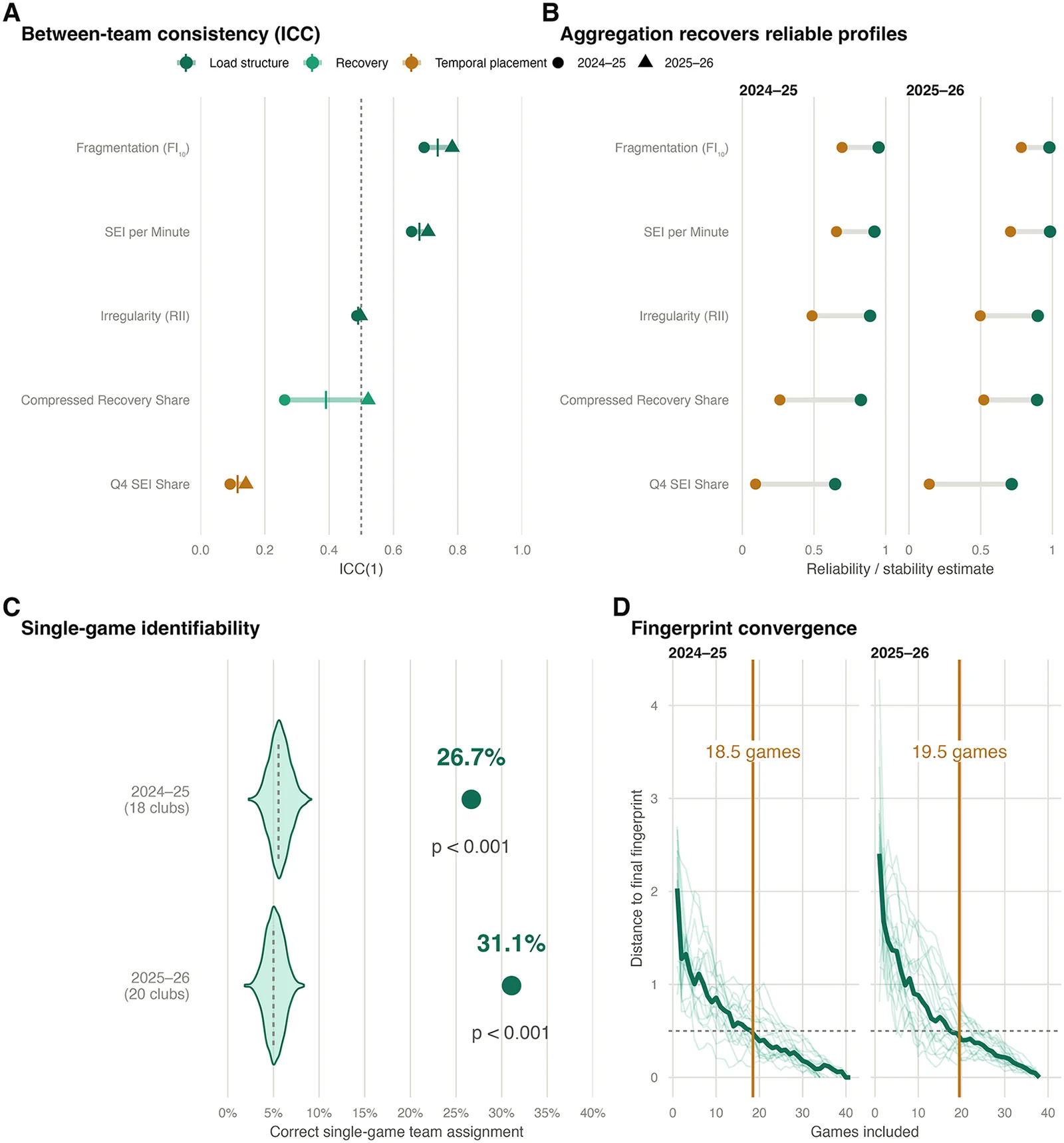
Within-season validity of the S5 signature. Panel A shows one-way random-effects ICC(1) for each game-level indicator. Panel B shows the gain from single-game consistency to aggregated split-half reliability. Panel C shows leave-one-out nearest-centroid identifiability against a permutation null. Panel D shows cumulative convergence of each club’s fingerprint toward its final season profile. All panels use only the five retained S5 indicators.

Low single-game consistency did not imply an unstable construct. Split-half analysis showed that game-level fluctuations aggregated into reliable seasonal profiles, with Spearman ρ = 0.65-0.95 in 2024−25 and 0.72-0.98 in 2025−26. The aggregation gain was largest precisely for Q4 SEI share, the indicator with the lowest single-game ICC (0.09 and 0.14), which nevertheless showed strong between-season stability (r = 0.74). Indicator-level descriptive and construct-validity statistics are reported in S6 and S7 Tables.

Games were assigned to their own club with 26.7% accuracy (95% CI [23.2, 30.0]) in 2024−25 (chance = 5.6%) and 31.1% (95% CI [27.8, 34.5]) in 2025−26 (chance = 5.0%) by leave-one-out nearest-centroid assignment. Permutation of club labels (B = 1,000) placed the observed accuracy beyond every permuted value (p < .001). A less restrictive distance criterion showed that 93.0% of games in 2024−25 and 97.0% in 2025−26 lay closer to their own club’s signature than to the average rival’s. Cumulative fingerprints stabilised within 0.5 standardised units of their season-final value after a median of 18.5 and 19.5 games; this figure is specific to the present two seasons and to the 0.5-unit criterion, and should be read as descriptive of this sample rather than as a general convergence benchmark.

### Team-season fingerprints form a coherent signature profile

Ordered by the structural consolidation score, the 38 team-seasons formed a continuum rather than discrete clusters, from Paris Basketball 2025 at the fragmented pole to Hapoel Tel Aviv 2025 at the consolidated pole (Fig 3). Žalgiris Kaunas 2025 occupied the extreme of the temporal-placement contrast, its signature defined by when it spent load rather than by its position on the consolidation continuum. The complete pairwise S5 distance matrix is shown descriptively in S1 Fig.

**Fig 3:**
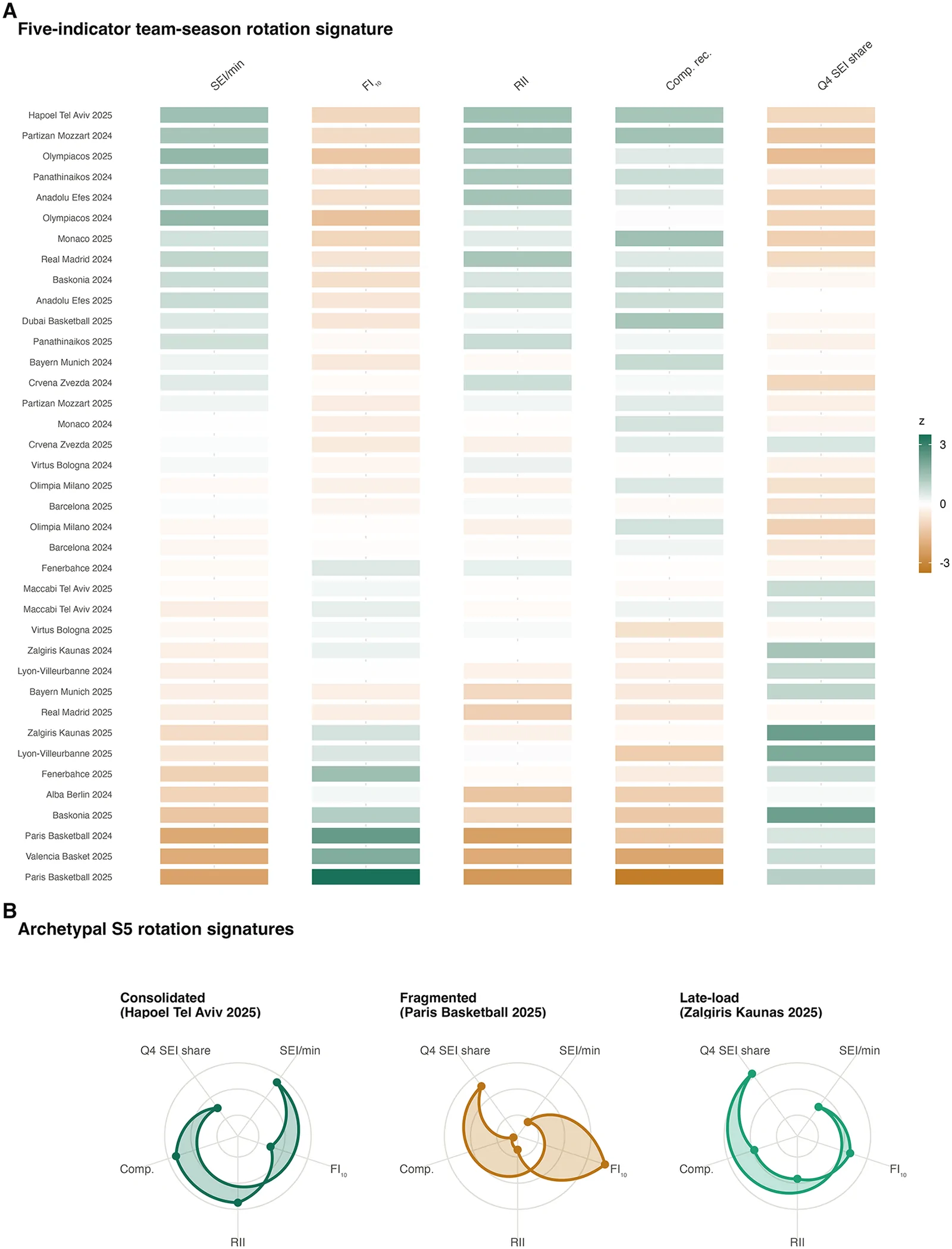
Team-season rotation signatures. Panel A shows the five retained indicators for each team-season, standardised and ordered by the predefined structural consolidation score. Panel B shows three archetypal profiles selected from the S5 geometry: consolidated, fragmented and late-load.

The five indicators were not independent dimensions. SEI per minute, fragmentation, irregularity and compressed recovery were strongly intercorrelated (maximum |r| = 0.92), and their covariation is substantive rather than artefactual: a club sustaining long stints necessarily shows greater dispersion in stint length and less complete recovery between them. Read together, they describe one coherent pattern of load structure rather than four separate constructs. Q4 SEI share supplies the contrast, adding temporal placement to that pattern. Correlations and variance inflation factors are documented in S8 Table.

### Rotation signatures persist across seasons

Seventeen clubs competed in both seasons. Matched clubs sat a median 1.55 standardised units from their own previous signature (IQR 0.96-2.03), spanning Olimpia Milano (0.52) to Baskonia (4.71; Fig 4). The 272 non-matching cross-season pairs were farther apart (median 2.20). Holding first-season profiles fixed and randomly reassigning the club correspondence of second-season profiles, no permutation produced a mean matched distance as small as the observed one (1.68 against a permutation mean of 2.62; p < .001).

**Fig 4:**
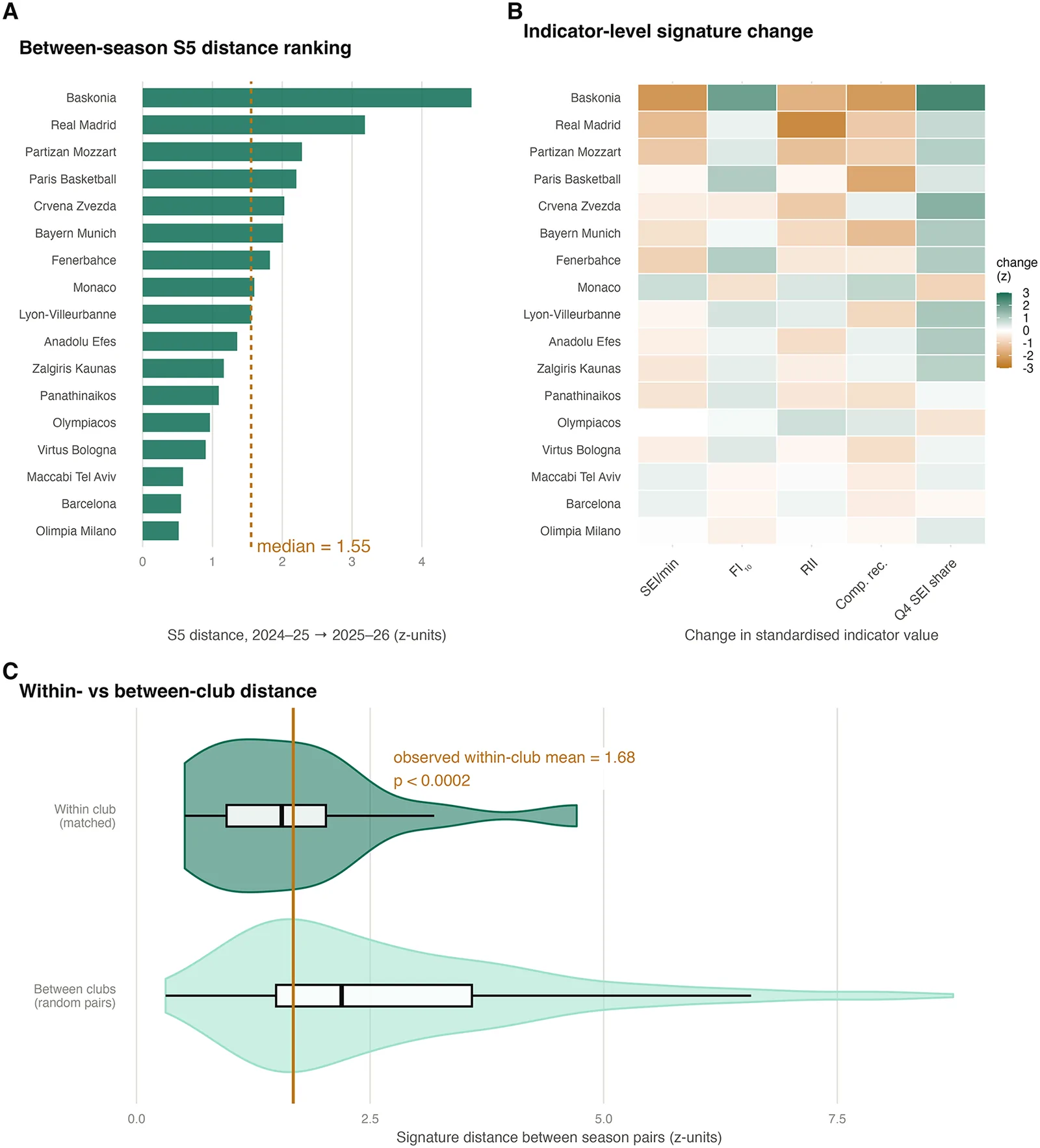
Between-season persistence of S5 rotation signatures. Panel A ranks clubs by the Euclidean distance between their 2024−25 and 2025−26 S5 fingerprints. Panel B decomposes each club’s signed indicator-level change. Panel C compares matched within-club distances with non-matching between-club distances and the permutation null. Distances are computed in the full five-dimensional standardised signature space.

The conclusion did not depend on the distance metric or on any single indicator. Under a regularised Mahalanobis metric, the observed mean matched distance was 1.71 against a permutation mean of 2.49 (p < .001), and each of the five leave-one-indicator-out subsets returned p < .001 (S9 Table).

Exploratory inspection of organisational continuity suggested larger S5 displacement among clubs that changed head coach between or during seasons. The 12 clubs with at least one transition had a median between-season distance of 1.80 standardised units (IQR 1.10-2.22) versus 1.09 (IQR 0.96-1.55) for the five clubs retaining the same coach, a difference of 0.71 standardised units that was imprecisely estimated (bootstrap 95% CI [−0.39, + 1.33]; exact permutation p = .241; exact Mann-Whitney p = .279). All six clubs with the largest displacement experienced a transition. Because coaching changes frequently coincided with roster and organisational changes, these findings are descriptive rather than causal (S5 Table). Table 3 summarises the convergent evidence across evidential scales.

**Table 3:** Summary of convergent evidence supporting the S5 rotation signature.

Evidential scale	Statistic	2024−25	2025−26	Inference
Single-game identifiability	LOO nearest-centroid accuracy	26.7% (chance 5.6%)	31.1% (chance 5.0%)	p < .001
Within-season reliability	Split-half Spearman ρ (range)	0.65-0.95	0.72-0.98	–
Between-season persistence	Matched vs random signature distance	–	–	mean = 1.68 vs null = 2.62; p <.001
Responsiveness to game state	Within-club variance explained by margin + congestion	–	–	signature 0.8% vs rotation depth 14.33%

### The game moves rotation depth far more than rotation architecture

If the signature were assembled in-game, the scoreboard and the calendar should move it. Across 1,366 team-games with club-season fixed effects, they moved it, but moved something else far more.

A 20-point margin entering the final quarter raised a club’s effective rotation by 1.03 standard deviations and the number of players used by 0.74; game state explained 14.33% of the within-club variance in effective rotation (Table 4). Neither quantity belongs to the signature. The same 20-point margin moved composite signature deviation by 0.24 standard deviations, and game state explained 0.80% of its within-club variance. Thus, the scoreboard accounted for roughly 18 times more variance in how many players a club used than in how it structured their exposure. Q4 SEI share did not respond to margin (t = −0.19; R² = 0.04%).

**Table 4:** Response of each rotation quantity to game state across all 1,366 team-games.

Quantity	In signature?	Δ per 20-point margin (SD)	t	R² within
Effective rotation	No	+1.03	+11.18	14.33
Players used	No	+0.74	+8.36	7.41
Compressed recovery	Yes	−0.49	−6.49	3.38
Fragmentation (FI10)	Yes	−0.40	−4.93	2.12
Irregularity (RII)	Yes	−0.32	−4.89	1.74
SEI per minute	Yes	−0.25	−3.09	1.00
Q4 SEI share	Yes	−0.02	−0.19	0.04
Signature deviation	Composite	+0.24	+2.57	0.80

Coefficients are changes in standard deviations produced by a 20-point margin entering the final quarter. R² is the within-club variance jointly explained by margin and congestion. Effective rotation and players used are diagnostic quantities outside the signature.

The signature indicators were not immune. Compressed recovery, fragmentation, irregularity and structural exposure per minute all responded significantly to margin, in the direction predicted by a bench-emptying account: blowouts lengthened stints, reduced fragmentation and relaxed recovery compression. But each moved between two and four times less than rotation depth, and each explained between one and three percent of within-club variance against fourteen percent for effective rotation. The claim is comparative, not absolute: rotation architecture is not invariant to the game; it is an order of magnitude less responsive to it than rotation depth.

Restricting the analysis to the 498 team-games still within five points entering the final quarter removed every effect, including the depth response. Game state explained 0.47% of within-club variance in signature deviation in these games, and no coefficient reached significance (S10 Table). The perturbation observed across the full sample was therefore concentrated in games with double-digit margins entering the final quarter. Among the 488 team-games separated by more than 10 points, margin significantly predicted signature deviation (t = 2.76; R² = 2.47%).

Schedule congestion was not detectable: across all games the coefficient on a congested second leg was −0.033 standardised units (95% CI [−0.145, + 0.079]), and the point estimate for composite signature deviation was negative in every specification. These are targeted perturbations. Opponent strength, venue, rest days and injury availability were not modelled; the analysis establishes that the scoreboard governs how many players a club uses far more than it governs how that club structures their exposure.

### Rotation architecture is not reducible to success, but temporal placement is not neutral

Structural consolidation, the unit-weight score summarising the dominant load-structure pattern, was not detectably associated with regular-season success. Across 38 team-seasons, the correlation with win percentage was r = +0.25; a cluster bootstrap resampling whole clubs returned a 95% interval of [−0.06, + 0.57], and a within-season permutation test gave p = .122. The score shared roughly 6% of the variance in winning. The dominant expression of a club’s rotation identity therefore appears to reflect style rather than competitive quality.

The temporal-placement dimension was not neutral. Clubs concentrating a larger share of their structural load in the fourth quarter won fewer games (r = −0.38 with win percentage; bootstrap 95% CI [−0.66, −0.05]; permutation p = .014) and posted worse point differentials (r = −0.39). The association accounted for 14.4% of the variance in win percentage and replicated almost exactly in each season considered separately (r = −0.41 in both seasons). Clubs in the bottom third of fourth-quarter SEI share won 58.3% of their games, whereas those in the top third won 43.9%, a difference of 14.4 percentage points, equivalent to approximately five wins across a regular season. This association is cross-sectional and based on a small number of team-seasons; it attenuated after controlling for previous-season success and is therefore reported as exploratory, not as evidence that temporal placement confers a quality-independent effect on outcomes. Associations between both signature dimensions and competitive outcomes are summarised in Table 5.

**Table 5:** Association between signature dimensions and regular-season success.

Signature dimension	Outcome	r	Bootstrap 95% CI	Perm. p	Shared variance
Structural consolidation	Win percentage	+0.253	[-0.056, +0.572]	.122	6.4%
Structural consolidation	Point differential	+0.291	[+0.008, +0.645]	–	8.5%
Q4 SEI share	Win percentage	−0.379	[-0.655, -0.045]	.014	14.4%
Q4 SEI share	Point differential	−0.392	[-0.699, -0.041]	–	15.4%

Intervals come from a cluster bootstrap resampling whole clubs (B = 5,000); permutation p-values are computed within season (B = 10,000). The Q4 association was not explained by exposure to decided games: the partial correlation with win percentage was −0.384 after controlling for share of decided games and −0.414 after controlling for mean absolute margin; Q4 SEI share correlated weakly with those controls (r = −0.082 and −0.099, respectively). A matched-club quality-control analysis using previous-season win percentage is reported in the text.

The obvious objection is reverse causation: a losing club may play the fourth quarter in earnest, while one winning comfortably rests its starters. Three lines of evidence argue against this. Q4 SEI share was the only signature indicator that did not respond to the margin entering the final quarter; between clubs, Q4 SEI share was essentially uncorrelated with exposure to games separated by more than 10 points entering the final period (r = −0.08) or with mean absolute margin (r = −0.10); and adjusting for either control left the association essentially unchanged. Among the 17 clubs present in both seasons, the partial correlation between 2025−26 Q4 SEI share and 2025−26 win percentage, controlling for the club’s 2024−25 win percentage, remained negative but was attenuated and imprecisely estimated (r = −0.20, bootstrap 95% CI [−0.68, + 0.34], p = .441).

Rotation architecture is therefore not reducible to competitive success: structural consolidation showed no detectable association with winning. The signature was not wholly independent of performance, however. Where a club placed its heaviest exposure within a game carried performance information that the structural organisation of that exposure did not.

## Discussion

### What the signature is, and what it is not

The principal finding is that substitution decisions leave a persistent organisational trace. Clubs alter personnel in response to the game, but retain a recognisable way of assembling competitive exposure.

Rotation depth and rotation architecture are different objects, and the results separate them cleanly. Basketball research has usually asked which players or line-ups are used, how combinations perform, or what happens immediately after a substitution [5,6,26,27]. Those questions are essential, but they do not describe how exposure is distributed through time. Two teams may both use nine players for similar total minutes and still manage the game very differently. One may leave its primary group on court for long uninterrupted stints, use a short bench bridge, and return the starters together; another may alternate players every three or four minutes and repeatedly reconfigure roles. Conventional minutes and line-up summaries can look similar, while the accumulated demands on continuity, recovery and late-game availability are not. S5 captures this second layer: the temporal grammar through which a club assembles playing exposure.

This distinction makes the game-state result easier to interpret in basketball terms. When the margin reaches 20 points before the final quarter, coaches predictably widen the rotation, protect key players and give peripheral players court time. Effective rotation therefore changes sharply. Yet the architecture changes far less. A coach may replace the starting point guard with the reserve, empty the bench, or protect a player in foul trouble, but the club still tends to organise the replacement minutes through its familiar pattern of stint length, fragmentation and recovery. The findings do not imply that substitutions are insensitive to the score; they show that the scoreboard changes who is used much more than it changes the club’s habitual way of structuring use. Rotation depth behaves as a situational decision of the kind documented across the situational-variable literature [10–13], whereas rotation architecture provides the stable reference against which that decision can be interpreted.

The indicator-admission rule strengthens that interpretation. Performance indicators are useful to staff only when they add information and remain sufficiently stable to describe the object of interest [1,2,4]. In practice, a dashboard becomes less informative when several variables are different labels for the same feature, or when a striking number is driven by rare game-specific events. Requiring each indicator to be both non-redundant and persistent removed both problems. The resulting signature is therefore not a collection of available substitution statistics; it is a deliberately restricted description of the dimensions that repeatedly distinguish clubs.

### Stable organisation without exact repetition

The psychometric pattern separates identity from repetition. Q4 SEI share had the lowest single-game consistency but strong between-season persistence: a team’s fourth-quarter burden is not reproduced game after game, yet some clubs repeatedly arrive at the final quarter with more structural load still to spend. Stability therefore appeared as a distributional tendency rather than the repetition of a fixed script, consistent with basketball as a self-organising system in which stable function emerges from variable local solutions [17,28,29].

The convergence result gives this a practical timescale. Roughly nineteen games were required before a club’s cumulative fingerprint settled close to its final profile, so a three-game run of shorter stints or compressed recovery should not be read as a new rotation policy; it may reflect injuries, foul trouble, travel or a sequence of decided games. A sustained displacement after the fingerprint stabilises is more credible evidence of real change. A club is legible from one game but only estimable from about twenty, giving normative profiling, long tied to the number of matches aggregated [3,4], an explicit multivariate convergence criterion at team level.

The 93–97% own-club proximity and the persistence analysis reinforce the same interpretation: line-ups, roles and availability may change, yet the club remains recognisable from how minutes are temporally assembled. This complements line-up and substitution-network research on the combinations teams reuse and the roles of core, connector and peripheral line-ups [6,26,27]. S5 adds the missing temporal dimension: line-up analysis identifies who shares the court, while rotation architecture describes how long combinations are sustained, how often the structure breaks, how much recovery follows, and when the burden is concentrated. Rotation depth is tactical; rotation architecture behaves as a comparatively persistent club-level property, a pattern that channels how game behaviour emerges without prescribing its realisations [15,16,19]. Comparable club-level regularities have been reported for playing styles in elite soccer, where teams retain identifiable stylistic profiles across matches despite contextual variation [30].

The exploratory coaching-continuity analysis gives a first indication of what may move that organisational trace. Clubs undergoing a head-coach transition showed larger median S5 displacement, and every club in the six largest shifts changed coach during the observation window; the uncertainty was nevertheless substantial, and several clubs retained stable fingerprints despite a transition. Rotation architecture should therefore not be read as the head coach’s signature alone, but as emerging from the interaction of several plausible determinants. These can be grouped into three families. Strategic and tactical determinants include head-coach philosophy, coaching-staff dynamics, the playing system and player positional versatility. Roster and structural determinants include squad composition, age profile, top-player availability, injury load and tactical match-ups. Organisational and environmental determinants include club philosophy and resourcing, schedule density and season objectives. The present design cannot separate these contributions, and doing so is a priority for future work. S5 may thus be stable enough to index organisational identity, yet sensitive enough to flag substantial reorganisation when that identity changes.

### Applied meaning: Diagnostic, not prescriptive

The practical value of the signature is diagnostic rather than prescriptive: consolidation was not detectably associated with winning, and no profile is inherently superior. Its value is making the operational consequences of a rotation policy visible. A player may finish 28 minutes as four balanced stints in one game and as two extended stints plus a short-rest fourth-quarter re-entry in another; a staff that believes it is ‘keeping the starters fresh’ may instead be imposing repeated short rests and compressed re-entry. S5 supplies a common language through which coaches, performance and medical staff can distinguish minutes played from how those minutes were experienced.

The five indicators can be translated directly into basketball questions. SEI per minute asks whether load is being accumulated through long, sequenced stints rather than through the same total minutes spread across shorter appearances. Fragmentation asks how frequently continuity is interrupted. RII asks whether players return in a predictable cadence or through highly variable stint sequences. Compressed recovery asks how often a player is asked to re-enter before receiving at least as much rest as the preceding stint; this matters because preliminary evidence indicates that prolonged bench inactivity reduces substitute readiness, making the timing of re-entry a preparation problem as well as a workload problem [31]. Q4 SEI share asks whether the main competitive burden has been distributed earlier or deferred to the closing period. None of these questions tells a coach whom to substitute, but together they reveal the operational consequences of the substitution plan.

Four uses follow. Opponent scouting gains a description of how exposure is assembled rather than a count of players used. Post-game review can separate an intended tactical adjustment from an abnormal architectural deviation. Season monitoring gains the ~ 19-game convergence point, which prevents short runs of unusual games being mistaken for structural change. Return-to-play and load monitoring gain a way to flag when nominally restricted minutes are delivered in demanding temporal configurations, consistent with evidence that match demands vary across playing periods and that accumulated load and congestion carry consequences for performance and injury risk [11,14,22]. These complement line-up-effectiveness models [27] by showing how combinations are deployed over the game.

The fourth-quarter result most tempts prescription, but its value is as a diagnostic question. A high Q4 SEI share may indicate a short trusted rotation, protection of primary creators for closing minutes, repeated late re-entry after short recovery, or dependence on a small group in close games. The association with winning replicated across seasons and was not explained by exposure to decided games, but weakened under the previous-season quality control. Staff should ask why the burden is deferred, who carries it, and whether that is planned or forced; the data do not show that mechanically reducing Q4 SEI share improves results.

### Limitations

Eight limitations bound these conclusions. First, the signature is defined over two regular seasons of one competition and does not include playoffs. Second, the indicators are conceptually distinct but empirically correlated (maximum |r| = 0.92), so the signature must be read as a coherent profile rather than as five independent measurements; conclusions were verified under a regularised Mahalanobis metric, which accounts for the covariance among indicators, and under removal of each indicator in turn (matched-club Mahalanobis distance 1.71 versus 2.49 expected by chance, p < .001 across 5,000 permutations). Third, persistence and invariance are established statistically, not causally: the design cannot separate club identity from coaching, roster and resource continuities that plausibly generate it. Fourth, the congested-second-leg indicator is a three-day proxy for the double-round rather than the competition’s own designation. Fifth, the Q4 SEI share association with success is cross-sectional and may reflect roster depth or bench quality rather than a causal effect of temporal load placement. Sixth, the coaching-continuity analysis was post hoc and based on only 17 matched clubs; head-coach transitions were also entangled with roster turnover, injuries and wider organisational change. It should therefore be treated as hypothesis-generating rather than as evidence of an independent coaching effect. Seventh, the five indicators were selected in part for their between-season stability, and the same two seasons were then used to demonstrate persistence; this makes the persistence analysis an internal validation of a proposed signature rather than an independent confirmation, and replication in further seasons and competitions is required before the S5 signature can be regarded as an established tool. Eighth, the Structural Exposure Index depends on parameter choices (class weights, exponents, a sequence penalty and a fourth-quarter multiplier) that were fixed a priori rather than tuned to outcomes; a sensitivity analysis showed that club separation in the S5 space was robust to these choices, including a fully linear index stripped of all weights, exponents and contextual multipliers (S1 Table), but the descriptive index values themselves are conditional on the chosen parameters.

## Conclusions

Elite basketball rotation is not exhausted by minutes, players used or substitution counts. Beneath rotation depth lies a comparatively stable architecture of competitive exposure, defined by how stints are loaded, fragmented, sequenced, recovered and temporally placed. It is visible from single games, becomes reliable through aggregation, persists across seasons, and is far less responsive to the scoreboard than rotation depth. This reframes substitution behaviour: coaches change who plays in response to the game, but the way a club structures exposure behaves more like a persistent property than a momentary tactic. Minutes played describe how much exposure was accumulated; rotation architecture describes how it was delivered, letting staff see whether an apparently ordinary rotation protected recovery, fragmented roles, or deferred responsibility to the final quarter. Because the fingerprint is stable under ordinary variation yet responsive to some organisational transitions, it may also offer an objective framework for monitoring how coaching, roster and strategic adaptation reshape exposure over time. The S5 signature offers a compact and interpretable framework for that purpose; having been evaluated within two seasons of a single competition, it is best regarded as a proposed signature that requires external replication before it can be treated as an established monitoring tool.

## Supporting information

S1 Dataset Minimal derived dataset. The aggregated, de-identified team-game and team-season indicator values underlying all figures, tables, and reported statistics, sufficient to reproduce the results reported in the manuscript.
(XLSX)

S1 Table Sensitivity of club identifiability to Structural Exposure Index parameters. Between-to-within club distance ratios in the five-indicator signature space under seven SEI specifications. A ratio above 1 indicates that team-seasons of the same club are closer to one another than to those of other clubs. The ratio remained between 1.33 and 1.69 across all specifications, including a fully linear index stripped of all weights, exponents and contextual multipliers, indicating that club separation does not depend on the SEI parameter choices.
(DOCX)

S2 Table Per-club single-game classification accuracy. Leave-one-out nearest-centroid correct-assignment rates for each club, ordered by accuracy within each season. Rates ranged from 0% to 100% and were structured rather than uniform: a minority of clubs carried highly distinctive rotation signatures (twelve of eighteen clubs above twice chance and seven above five times chance in 2024−25), while others were indistinguishable from the league norm. Club distinctiveness was itself persistent across seasons. Overall accuracy was 26.7% (2024−25) and 31.1% (2025−26) against chance levels of 5.6% and 5.0%.
(DOCX)

S3 Table Crossed random-effects repeatability of the signature indicators. Variance components from a linear mixed model fitted to each standardised indicator with club and season as crossed random intercepts, and the implied between-club repeatability (club variance divided by the total of club, season and residual variance). Repeatability was substantial for four of the five indicators and lowest for fourth-quarter SEI share, with a five-indicator mean of 0.44.
(DOCX)

S4 Table Identifiability of reduced representations. Leave-one-out nearest-centroid single-game classification accuracy for the full five-indicator signature compared with three reduced representations, by season and averaged, against nominal chance. Rotation depth alone classified games barely above chance, whereas the full signature was the most identifiable, indicating that rotation architecture carries club-identifying information that rotation depth does not.
(DOCX)

S5 Table Exploratory head-coach continuity audit for the 17 clubs present in both seasons. S5 distance is the Euclidean distance between each club’s 2024−25 and 2025−26 standardised fingerprints.
(DOCX)

S6 Table Indicator-level descriptive statistics and reliability estimates at team-game scale, by season. ICC(1) is the one-way random-effects intraclass correlation across clubs; split-half reliability is the Spearman rank correlation between odd- and even-game indicator means across clubs.
(DOCX)

S7 Table Construct validity of the five retained indicators. The dissociation between single-game consistency and between-season stability for fourth-quarter SEI share is the central psychometric result.
(DOCX)

S8 Table Correlation structure and multicollinearity of the retained S5 indicators (n = 38 team-seasons). The signature is intentionally not orthogonal: load-structure indicators form a coherent pattern, while Q4 SEI share adds a temporal-placement contrast. Variance inflation factors document collinearity; no latent components or component scores are defined.
(DOCX)

S9 Table Sensitivity of the between-season persistence test to the distance metric and to indicator removal. B = 5,000 permutations, seed 20260706, throughout.
(DOCX)

S10 Table Invariance to game state, restricted to games still within five points entering the fourth quarter (n = 498). Coefficients and intervals are in standard deviations of the outcome; R² is the within-club variance jointly explained by margin and second-leg congestion.
(DOCX)

S1 Fig Pairwise S5 distance matrix. Euclidean distances are computed in the full five-dimensional standardised signature space. Rows and columns follow the structural-consolidation order. The matrix is descriptive; inference uses matched-versus-random distance tests.
(PNG)
