# Supplementary material for: Beyond minutes played: Rotation architecture as a persistent club-level signature in elite basketball
Source: PLoS One. 2026 Oct 27;21(10):e0360790. doi: 10.1371/journal.pone.0360790 (PMC13712522; doi:10.1371/journal.pone.0360790)
Supplement: Supplementary file 1 [file pone.0360790.s002.docx]

**S1 Table**

| **SEI specification** | **Between-/within-club distance ratio** |
| --- | --- |
| Baseline (published index) | 1.64 |
| Fully linear (no weights, exponents, or context) | 1.33 |
| No contextual multipliers | 1.64 |
| Class thresholds -1 min | 1.69 |
| Class thresholds +1 min | 1.65 |
| Flattened exponents | 1.61 |
| Stronger Q4 multiplier (1.15) | 1.64 |
