# Supplementary material for: Beyond minutes played: Rotation architecture as a persistent club-level signature in elite basketball
Source: PLoS One. 2026 Oct 27;21(10):e0360790. doi: 10.1371/journal.pone.0360790 (PMC13712522; doi:10.1371/journal.pone.0360790)
Supplement: Supplementary file 2 [file pone.0360790.s003.docx]

**S2 Table**

| **Rank** | **Club** | **Accuracy** | **Games** | **Club** | **Accuracy** | **Games** |
| --- | --- | --- | --- | --- | --- | --- |
|  | ***2024-25 (chance 5.6%)*** | | | ***2025-26 (chance 5.0%)*** | | |
| 1 | PRS | 100.0% | 38 | PRS | 78.9% | 38 |
| 2 | OLY | 57.5% | 40 | VAL | 76.3% | 38 |
| 3 | BER | 51.5% | 33 | OLY | 55.3% | 38 |
| 4 | MIL | 44.1% | 34 | BAS | 47.4% | 38 |
| 5 | PAR | 41.2% | 34 | HTA | 47.4% | 38 |
| 6 | PAN | 30.0% | 40 | MAD | 42.1% | 38 |
| 7 | ZAL | 29.4% | 34 | ULK | 39.5% | 38 |
| 8 | IST | 23.7% | 38 | ASV | 31.6% | 38 |
| 9 | RED | 20.0% | 35 | MCO | 30.6% | 36 |
| 10 | BAY | 16.7% | 36 | ZAL | 28.9% | 38 |
| 11 | ULK | 15.4% | 39 | PAN | 21.6% | 37 |
| 12 | TEL | 15.2% | 33 | DUB | 21.1% | 38 |
| 13 | ASV | 8.8% | 34 | MIL | 21.1% | 38 |
| 14 | BAR | 7.7% | 39 | TEL | 18.9% | 37 |
| 15 | MCO | 7.3% | 41 | IST | 15.8% | 38 |
| 16 | BAS | 5.9% | 34 | RED | 15.8% | 38 |
| 17 | MAD | 5.0% | 40 | VIR | 15.8% | 38 |
| 18 | VIR | 0.0% | 34 | BAY | 10.5% | 38 |
| 19 |  |  |  | BAR | 2.6% | 38 |
| 20 |  |  |  | PAR | 0.0% | 38 |
