# Supplementary material for: Beyond minutes played: Rotation architecture as a persistent club-level signature in elite basketball
Source: PLoS One. 2026 Oct 27;21(10):e0360790. doi: 10.1371/journal.pone.0360790 (PMC13712522; doi:10.1371/journal.pone.0360790)
Supplement: Supplementary file 3 [file pone.0360790.s004.docx]

**S3 Table**

| **Indicator** | **Club variance** | **Season variance** | **Residual variance** | **Between-club repeatability** |
| --- | --- | --- | --- | --- |
| Structural exposure per minute | 0.69 | 0.07 | 0.39 | 0.60 |
| Fragmentation (FI10) | 0.72 | 0.04 | 0.31 | 0.68 |
| Rotation irregularity (RII) | 0.49 | 0.05 | 0.58 | 0.44 |
| Compressed recovery | 0.45 | 0.06 | 0.63 | 0.39 |
| Fourth-quarter SEI share | 0.11 | 0.03 | 0.88 | 0.11 |
