# Supplementary material for: Beyond minutes played: Rotation architecture as a persistent club-level signature in elite basketball
Source: PLoS One. 2026 Oct 27;21(10):e0360790. doi: 10.1371/journal.pone.0360790 (PMC13712522; doi:10.1371/journal.pone.0360790)
Supplement: Supplementary file 4 [file pone.0360790.s005.docx]

**S4 Table**

| **Model (indicators)** | **2024-25** | **2025-26** | **Mean** |
| --- | --- | --- | --- |
| Full S5 signature (5 indicators) | 26.7% | 31.1% | 28.9% |
| Exposure/min + fragmentation (2) | 25.5% | 26.5% | 26.0% |
| Structural + temporal (2) | 19.1% | 19.0% | 19.1% |
| Rotation depth only (1) | 8.1% | 9.7% | 8.9% |
| Chance | 5.6% | 5.0% | 5.3% |
