# Supplementary material for: Beyond minutes played: Rotation architecture as a persistent club-level signature in elite basketball
Source: PLoS One. 2026 Oct 27;21(10):e0360790. doi: 10.1371/journal.pone.0360790 (PMC13712522; doi:10.1371/journal.pone.0360790)
Supplement: Supplementary file 5 [file pone.0360790.s006.docx]

**S5 Table**

| **Club** | **2024-25 head-coach sequence** | **2025-26 head-coach sequence** | **Transition timing** | **S5 distance** |
| --- | --- | --- | --- | --- |
| Baskonia | Pablo Laso | Paolo Galbiati | Between seasons | 4.71 |
| Real Madrid | Chus Mateo | Sergio Scariolo | Between seasons | 3.19 |
| Paris Basketball | Tiago Splitter | Francesco Tabellini → Julius Thomas (interim) | Between seasons | 2.28 |
| Partizan | Željko Obradović | Željko Obradović → Joan Peñarroya (after interim) | During 2025-26 | 2.20 |
| Crvena Zvezda | Ioannis Sfairopoulos | Ioannis Sfairopoulos → Saša Obradović | During 2025-26 | 2.03 |
| FC Bayern Munich | Gordon Herbert | Gordon Herbert → Svetislav Pešić | During 2025-26 | 2.01 |
| Fenerbahçe | Šarūnas Jasikevičius | Šarūnas Jasikevičius | Full continuity | 1.82 |
| AS Monaco | Saša Obradović → Vassilis Spanoulis | Vassilis Spanoulis → Manuchar Markoishvili | During 2025-26 | 1.60 |
| ASVEL | Pierric Poupet | Pierric Poupet | Full continuity | 1.55 |
| Anadolu Efes | Tomislav Mijatović → Luca Banchi | Igor Kokoškov → Pablo Laso | Between seasons | 1.35 |
| Žalgiris Kaunas | Andrea Trinchieri | Tomas Masiulis | Between seasons | 1.16 |
| Panathinaikos | Ergin Ataman | Ergin Ataman | Full continuity | 1.09 |
| Olympiacos | Georgios Bartzokas | Georgios Bartzokas | Full continuity | 0.96 |
| Virtus Bologna | Luca Banchi → Duško Ivanović | Duško Ivanović → Nenad Jakovljević | During 2025-26 | 0.90 |
| Maccabi Tel Aviv | Oded Kattash | Oded Kattash | Full continuity | 0.58 |
| FC Barcelona | Joan Peñarroya | Joan Peñarroya → Xavi Pascual | During 2025-26 | 0.55 |
| Olimpia Milano | Ettore Messina | Ettore Messina → Peppe Poeta | During 2025-26 | 0.52 |

Note. Coaching sequences document the head coaches contributing to each season-level fingerprint. A transition was coded when the head coach differed between seasons or changed during 2025-26. Clubs with any transition had a median S5 distance of 1.80 standardised units (IQR 1.10-2.22; n = 12), compared with 1.09 (IQR 0.96-1.55; n = 5) under full continuity. The difference was exploratory and imprecise (bootstrap 95% CI for the median difference [-0.39, +1.33]; exact permutation p = .241; exact Mann-Whitney p = .279). Coaching transitions may coincide with roster and wider organisational changes.
