# Supplementary material for: Beyond minutes played: Rotation architecture as a persistent club-level signature in elite basketball
Source: PLoS One. 2026 Oct 27;21(10):e0360790. doi: 10.1371/journal.pone.0360790 (PMC13712522; doi:10.1371/journal.pone.0360790)
Supplement: Supplementary file 6 [file pone.0360790.s007.docx]

**S6 Table**

| **Indicator** | **Season** | **M (SD)** | **ICC(1)** | **Split-half Spearman ρ** |
| --- | --- | --- | --- | --- |
| Fragmentation (FI10) | 2024-25 | 2.263 (0.372) | 0.696 | 0.950 |
| Fragmentation (FI10) | 2025-26 | 2.379 (0.487) | 0.783 | 0.979 |
| SEI per minute | 2024-25 | 406.085 (120.959) | 0.656 | 0.922 |
| SEI per minute | 2025-26 | 367.338 (127.578) | 0.708 | 0.983 |
| Irregularity (RII) | 2024-25 | 0.1821 (0.4437) | 0.486 | 0.891 |
| Irregularity (RII) | 2025-26 | 0.0611 (0.4438) | 0.497 | 0.898 |
| Compressed recovery | 2024-25 | 0.672 (0.096) | 0.262 | 0.827 |
| Compressed recovery | 2025-26 | 0.642 (0.115) | 0.521 | 0.893 |
| Q4 SEI share | 2024-25 | 0.279 (0.074) | 0.092 | 0.647 |
| Q4 SEI share | 2025-26 | 0.297 (0.088) | 0.141 | 0.716 |

Note. M and SD are computed at team-game scale before season-level aggregation. Indicators were standardised before multivariate distance calculations. RII is reported to four decimals because its season-specific SDs were nearly identical at three decimals.
