# Supplementary material for: Beyond minutes played: Rotation architecture as a persistent club-level signature in elite basketball
Source: PLoS One. 2026 Oct 27;21(10):e0360790. doi: 10.1371/journal.pone.0360790 (PMC13712522; doi:10.1371/journal.pone.0360790)
Supplement: Supplementary file 7 [file pone.0360790.s008.docx]

**S7 Table**

| **Indicator** | **ICC 24-25** | **ICC 25-26** | **ICC mean** | **Between-season r** | **Interpretation** |
| --- | --- | --- | --- | --- | --- |
| Fragmentation (FI10) | 0.696 | 0.783 | 0.740 | 0.84 | highly club-persistent |
| SEI per minute | 0.656 | 0.708 | 0.682 | 0.71 | highly club-persistent |
| Irregularity (RII) | 0.486 | 0.497 | 0.491 | 0.59 | moderately club-persistent |
| Compressed recovery | 0.262 | 0.521 | 0.392 | 0.64 | moderately club-persistent |
| Q4 SEI share | 0.092 | 0.141 | 0.116 | 0.74 | game-variable but season-persistent |
