# Supplementary material for: Beyond minutes played: Rotation architecture as a persistent club-level signature in elite basketball
Source: PLoS One. 2026 Oct 27;21(10):e0360790. doi: 10.1371/journal.pone.0360790 (PMC13712522; doi:10.1371/journal.pone.0360790)
Supplement: Supplementary file 8 [file pone.0360790.s009.docx]

**S8 Table**

| **Indicator** | **SEI/min** | **FI₁₀** | **RII** | **Comp** | **Q4** |
| --- | --- | --- | --- | --- | --- |
| SEI/min | 1.00 | -0.91 | 0.92 | 0.83 | -0.71 |
| FI10 | -0.91 | 1.00 | -0.78 | -0.84 | 0.63 |
| RII | 0.92 | -0.78 | 1.00 | 0.80 | -0.58 |
| Comp | 0.83 | -0.84 | 0.80 | 1.00 | -0.62 |
| Q4 | -0.71 | 0.63 | -0.58 | -0.62 | 1.00 |

*Variance inflation factors (each indicator regressed on the other four):*

| **Indicator** | **R²** | **VIF** |
| --- | --- | --- |
| SEI per minute | 0.956 | 22.7 |
| Irregularity (RII) | 0.897 | 9.7 |
| Fragmentation (FI10) | 0.885 | 8.7 |
| Compressed recovery | 0.770 | 4.4 |
| Q4 SEI share | 0.576 | 2.4 |
