# Supplementary material for: Beyond minutes played: Rotation architecture as a persistent club-level signature in elite basketball
Source: PLoS One. 2026 Oct 27;21(10):e0360790. doi: 10.1371/journal.pone.0360790 (PMC13712522; doi:10.1371/journal.pone.0360790)
Supplement: Supplementary file 9 [file pone.0360790.s010.docx]

**S9 Table**

| **Specification** | **Observed mean** | **Permutation mean** | **p** |
| --- | --- | --- | --- |
| Full S5 | 1.677 | 2.624 | < .001 |
| - SEI per minute | 1.543 | 2.361 | < .001 |
| - Fragmentation | 1.575 | 2.369 | < .001 |
| - Irregularity | 1.485 | 2.350 | < .001 |
| - Compressed recovery | 1.442 | 2.371 | < .001 |
| - Q4 SEI share | 1.404 | 2.189 | < .001 |
| Mahalanobis (λ = 0.10) | 1.713 | 2.492 | < .001 |
