# Supplementary material for: Beyond minutes played: Rotation architecture as a persistent club-level signature in elite basketball
Source: PLoS One. 2026 Oct 27;21(10):e0360790. doi: 10.1371/journal.pone.0360790 (PMC13712522; doi:10.1371/journal.pone.0360790)
Supplement: Supplementary file 10 [file pone.0360790.s011.docx]

**S10 Table**

| **Quantity** | **Δ per point** | **t** | **95% CI** | **Second leg** | **Second leg t** | **Second leg 95% CI** | **R²** |
| --- | --- | --- | --- | --- | --- | --- | --- |
| Effective rotation | +0.030 | +1.04 | [-0.027, +0.088] | -0.103 | -1.10 | [-0.287, +0.081] | 0.39% |
| Players used | +0.019 | +0.57 | [-0.045, +0.082] | -0.006 | -0.05 | [-0.210, +0.199] | 0.08% |
| Fragmentation (FI10) | +0.017 | +0.52 | [-0.045, +0.078] | -0.019 | -0.18 | [-0.225, +0.188] | 0.07% |
| SEI per minute | -0.030 | -0.90 | [-0.097, +0.036] | +0.115 | +1.09 | [-0.091, +0.321] | 0.44% |
| Irregularity (RII) | -0.016 | -0.57 | [-0.073, +0.040] | +0.119 | +0.99 | [-0.115, +0.353] | 0.31% |
| Compressed recovery | -0.020 | -0.71 | [-0.076, +0.036] | +0.095 | +1.13 | [-0.069, +0.258] | 0.25% |
| Q4 SEI share | +0.009 | +0.30 | [-0.049, +0.067] | +0.006 | +0.06 | [-0.214, +0.226] | 0.02% |
| Signature deviation | +0.025 | +0.78 | [-0.037, +0.087] | -0.138 | -1.28 | [-0.350, +0.074] | 0.47% |
